# Supplementary material for: Defining the genetic and evolutionary architecture of alternative splicing in response to infection
Source: Nat Commun. 2019 Apr 11;10:1671. doi: 10.1038/s41467-019-09689-7 (PMC6459842; doi:10.1038/s41467-019-09689-7)
Supplement: Supplementary file 1 — Supplementary Information [file 41467_2019_9689_MOESM1_ESM.pdf]

Defining the genetic and evolutionary architecture of alternative splicing in response to infection

Rotival et al.

Supplementary Note 1: Estimating the phylogenetic age of splice sites. To date the first occurrence in the phylogeny (phylogenetic age) of the splice sites present in the genome of modern humans, we retrieved MULTIZ-46way alignments from UCSC[1] and extracted orthologous sequences in a 100 bp window around each splice site (50 bp on each side). We then reconstructed the ancestral sequences by maximum likelihood, using the *ancestral_inference* script from the *treetime* software (ref.[2], available at https://github.com/neherlab/treetime). We next dated the emergence of the splice site based on its presence/absence in the ancestral sequence across the genealogy from humans to chordates. The following divergence times were assumed based on the literature[3]: Human-Chimp: 5.5 MY, Human-Gorilla: 6.7 MY, Human-Orangutans: 8.2 MY, Human-Gibbons: 14.6MY, Human-Macaque: 23.3 MY, Human-Shrews: 85 MY, Human-Mice: 90.8 MY, Human-Cow: 92 MY, Human-Elephant: 105 MY, Human-Opossum: 173 MY, Human-Frog: 360 MY, Human-Zebrafish: 450 MY, Human-Lamprey 564 MY. We calibrated the missing divergence times based on phylogenetic distances leading to the following estimates: Human-Tarsier: 65.3 MY, Human-Lemur: 73.7 MY, Human-Platypus: 211.9 MY, Human-Chicken: 270.8 MY.

Supplementary Note 2: Correction for technical variability. To account for technical variability in splicing quantification, we assessed the impact on PSI values of potential confounding factors, by fitting a mixed model for each AS event. Namely, we transformed PSI using a logit transformation $\left\{x \longrightarrow \log\left(\frac{x}{1-x}\right)\right\}$ and modelled the transformed values as a function of stimulation condition, population, and technical covariates. These included total RNA concentration, RIN, percentage of high-quality bases (Q30), mean GC content, library concentration, 5'/3' coverage bias (measured as the mean difference in coverage between the 5' and 3' ends of the gene) as continuous covariates, and date of experiment, library

preparation batch, sequencing batch, sequencer used, sequencing index, and sequencing lane as putative batch effects. Putative batch effects were modelled as random effects, and all other covariates (stimulation condition, population and continuous covariates) were included as fixed effects, leading to the following model for splicing event i and sample j:

$$\log\left(\frac{\tilde{\psi}_{ij}}{1-\tilde{\psi}_{ij}}\right) = \alpha + \beta_{cond} + \beta_{pop} + \sum_{c} \beta_c \text{Covariate}_c + \sum_{k} \beta_{k,j}^{\text{batch}} + \varepsilon_{ij} \quad (1)$$

where α is the intercept, β_{cond} and β_{pop} are the fixed effect of the condition and population on sample j, β_c are the fixed effect of continuous covariates on sample j, the $\beta_{k,j}^{\text{batch}}$ are the random effects of batch covariate k, on sample j, ε_{ij} are the residuals and $\tilde{\psi}_{ij}$ are rescaled PSI values computed as $\tilde{\psi}_{ij} = \delta + (1-\delta) * PSI_{ij}$ with $\delta > 0$ set to ensure that $0 < \tilde{\psi}_{ij} < 1$. In practice, we used $\delta = 0.001$.

The proportion of AS events affected by each factor is reported in **Supplementary Fig. 2f**, for various levels of variance explained. Date of the experiment, library batch, sequencer used, sequencing batch, GC content, and 5'/3' bias were detected as the strongest confounding factors. Accordingly, we corrected the data for these factors before analysis by sequentially running ComBat[4] on logit-transformed PSI values for each batch effect, with condition and population as covariates. Similarly, we adjusted for GC-content and 5'/3' bias, using linear models on logit–scale batch-corrected PSI values. Refitting our linear mixed model confirmed that correction was satisfactory for most of the technical covariates (**Supplementary Fig. 2f**).

Supplementary Note 3: Likelihood-based Causal Model Selection Framework. We used a two-step approach to establish causality between gene expression and splicing. First, we assessed the partial correlation between splicing and expression, adjusting for the genotype of the sQTL, and excluded cases where splicing is independent from gene expression (i.e., these

loci were classified as independent). Second, for the remaining loci, i.e., those where splicing and gene expression were not independent, we used a Likelihood-based Causal Model Selection Framework[5] to establish causality. Briefly, in this approach, which is related in its principle to Mendelian randomization, genetic variants are used as instrument variables to assess the causal impact of a covariate (typically, a biomarker) on a trait or disease of interest. Since genetic variants are randomized by meiotic recombination, correlations between genetics and phenotypic traits are unlikely to be affected by unwanted confounders, provided that population structure is adequately considered. Thus, one can resolve the causality between two correlated traits that are genetically controlled, here, gene expression and splicing, by comparing the likelihood of each causal path between genotype and the trait of interest.

Each possible causality scenario (splicing → expression or expression → splicing) can be linked to a specific conditional independence structure between genotype, splicing and gene expression (**Supplementary Fig. 6**):

- If splicing is mediating the changes in gene expression (splicing → expression, referred to as causal sQTL), we expect expression to be independent from genotype, once splicing is accounted for. For a genetic variant G showing significant association to gene expression E and splicing levels S, the likelihood of the model can thus be decomposed as follows:

$$L_1 = L(E, S \mid G, \text{Model 1}) = L(E|S).\ L(S|G) \qquad (2)$$

- Conversely, if gene expression is mediating the changes in splicing (expression → splicing, referred to as reactive sQTL), we expect splicing to be independent from genotype, once expression is accounted for. The likelihood of the model will thus be decomposed as:

$$L_2 = L(E, S \mid G, \text{Model 2}) = L(S|E).\ L(E|G) \qquad (3)$$

In both instances, the likelihoods L(E|S), L(S|G), L(S|E), L(E|G) can be obtained by applying a linear model to values of E and S and the minor allele counts of G. Namely, for a quantitative variable Y and a quantitative or binary variable X, we decompose Y as $Y = \alpha + \beta X + \gamma Pop + \varepsilon$, where Pop is a binary variable indicating African/European descent, α is the intercept of the model, β is the effect of X on Y, γ is the effect of population on Y, and ε is a normally distributed error term with variance σ^2. For n observations, the likelihood can then be written as:

$$L(Y|X) = \phi(Y; \alpha + \beta X, \sigma^2) = \frac{1}{(2\pi\sigma)^{n/2}} e^{-\frac{\|Y-(\alpha+\beta X+\gamma Pop)\|^2}{2\sigma^2}} \quad (4)$$

After computing the likelihoods, a probability can be assigned to each model as follows:

$$\text{Prob(Model j)} = L_j/(L_1 + L_2), \text{ for j} = 1,2. \quad (5)$$

We then retain the model with the highest probability as the most likely causal model.

Supplementary Note 4: Sharing of sQTLs across conditions. To assess the sharing of sQTLs across conditions, we focused on sQTLs of constitutively expressed genes (FPKM > 10 in all conditions) and tested, for each sQTL, the association between the peak SNP and the PSI across all conditions. We then used a Bayesian model selection approach to identify the specific and shared effects of the 1,464 detected sQTLs across the 5 conditions. Namely, for each sQTL and condition j, we defined indicator variables (γ_j) as:

$$\gamma_j = \begin{cases} 1 \text{ if the sQTL has an effect in condition j} \\ 0 \text{ otherwise} \end{cases} \quad (6)$$

For each sQTL, we then assessed the 32 models corresponding to all possible values of ($\gamma_.$) and assigned a likelihood to each model. Specifically, for each value of ($\gamma_.$), we modeled the PSI ψ_{ij} as a function of the genotype (SNP_i) of individual i, the population to which he belongs (Pop_i) and the condition of stimulation j, under the following model:

$$\psi_{ij} = \alpha_j + \beta \cdot \gamma_j \cdot SNP_i + \delta_j \cdot Pop_i + \varepsilon_{ij} \quad (7)$$

where α_j is the intercept for condition j, β is the mean effect of one allele of the sQTL across the conditions in which it is active, δ_j is the effect of population on ψ_{ij} in condition j, and ε_{ij} is a normally distributed noise. We then assigned the probability of each model as:

$$Prob(\text{Model } m) = \frac{Likelihood_m}{\sum_{k=1}^{32} Likelihood_k} \qquad (8)$$

Finally, the number of conditions in which the sQTL is active was obtained as $\sum_j \gamma_j$, based on the γ values from the most likely model.

Supplementary Note 5: Detection of Neanderthal introgressed sQTLs. To identify splice regulatory variants that were introduced into European genomes by archaic admixture, we first defined a list of 197,759 SNPs of putative Neanderthal origin, termed archaic SNPs (aSNPs). Briefly, the complete genome sequence of a Neanderthal individual from Altai[6] was used to identify genetic variants of likely Neanderthal origin that segregate in modern humans. The following criteria were used (see also ref.[7]): (i) one of the alleles is fixed in the Neanderthal and absent from the Yoruba population of the 1000 Genomes phase 3 (ref.[8]), (ii) the Neanderthal allele is present in one of the 100 European individuals from the EvoImmunoPop cohort[7], and (iii) the putative archaic variant overlaps a region of the modern human genome where Neandertal ancestry has been predicted with a high degree of confidence (marginal probability of Neandertal ancestry > 0.9 and genetic length > 0.02 cM)[9]. Among the resulting set of 197,759 aSNPs, 36,618 aSNPs presented a MAF ≥ 5% in our setting (i.e., MAF ≥ 10% in Europeans). For each of these, we measured the boundaries of the haplotype on which it is located by extracting all aSNPs in high LD ($r^2 > 0.8$) with the target aSNP. Once excluding variants laying on a haplotype shorter than 10 kb, to limit cases of incomplete lineage sorting, we obtained a final set of 31,954 high-confidence aSNPs. We then extended this list to include 68,801 additional SNPs in high LD ($r^2 > 0.8$) with an aSNP (tag-

aSNPs). This allowed us to capture variants that pre-existed in the human lineage before admixture with Neanderthal (i.e., present in Africans) but were lost in Europeans before being reintroduced through admixture with Neanderthal (i.e., these variants are found exclusively on Neanderthal haplotypes in Europe). This led to a final set of 100,755 frequent SNPs (MAF ≥ 5%) that are exclusively present on Neanderthal haplotypes in Europe. To assess the robustness of the enrichments we report to changes in the definition of Neanderthal introgressed haplotypes, we repeated these enrichments for increasing haplotype lengths (20 kb, 30 kb and 50 kb) and minimal number of aSNPs per haplotype (2, 5 or 10), and obtained similar results (see **Supplementary Data 4)**.

Supplementary Note 6: Evaluating the purity of the monocyte fraction. When purifying monocytes based on CD14 beads, one major concern is the ability to efficiently separate monocytes from $CD14^{+}$-expressing neutrophils. To exclude a possible contamination of the monocyte populations by neutrophils, we performed flow cytometry analysis using a panel of four antibodies (i.e., CD15, CD66b, CD16 and CD14) in order to characterise monocyte and neutrophil populations. Neutrophils are defined as $CD15^{+}$, $CD66b^{+}$, $CD16^{+}$ and $CD14^{low}$ (**Supplementary Fig. 8**), whereas monocytes, which are $CD15^{-}$ and $CD66b^{-}$, are composed of $CD14^{high}/CD16^{-}$ classical monocytes, $CD14^{high}/CD16^{+}$ intermediate monocytes, and $CD14^{low}/CD16^{+}$ non-classical monocytes. We obtained purified $CD14^{+}$ monocytes from whole blood, using the same experimental settings as in Quach *et al.* 2016 (ref.[7]), and used the granulocyte/erythrocyte layer obtained from a whole blood density gradient centrifugation as a positive control for neutrophils. Our data confirmed that, with our protocol, neutrophils are virtually absent from the purified $CD14^{+}$ monocyte cell fraction (<0.12%), whereas they are, as expected, at high proportions in the granulocyte/erythrocyte layer (**Supplementary Figure 8)**.

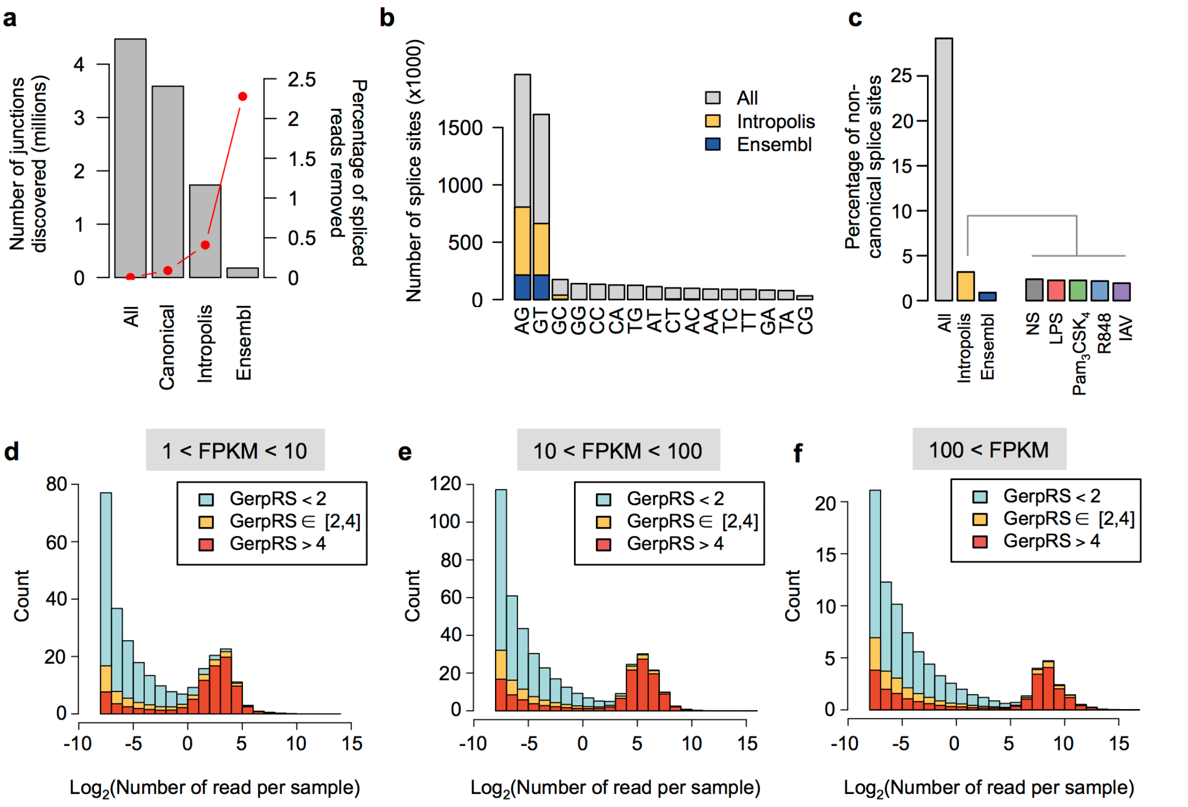

Supplementary Figure 1 | Quality control of detected splice sites and relationship between conservation and activity.

a Number of observed junctions (grey bars) and percentage of spliced reads that are removed (red dots) after each filtering criteria. All: no filtering; Canonical: only reads where the donor-acceptor sites match the canonical GT-AG, GC-AG or AT-AC sequences; intropolis: only spliced reads that map to known genic regions and match introns from the intropolis database; Ensembl: only spliced reads where both ends map on an exon annotated in Ensembl v70. **b** Distribution of di-nucleotides at donor/acceptor sites inferred from spliced reads, when considering all splice sites (grey), splice sites present in the intropolis database (yellow), or splice sites present in Ensembl database (blue). **c** Percentage of AG/GT splice sites for each of the previous filters. For splice sites present in intropolis, we further report this percentage, for each condition separately. **d-f** Conservation as a function of splice site activity. For each bin of gene expression (panel **d,** 1 < FPKM < 10; panel **e,** 10 < FPKM <100; panel **f**, FPKM > 100), histograms show the distribution of splice site activity, measured by the average number of supporting reads per sample. Within each bin, colours indicate the fraction of splice sites that are highly conserved (GerpRS > 4, red), moderately conserved (2 < GerpRS < 4, yellow) or non-conserved (GerpRS < 2, blue).

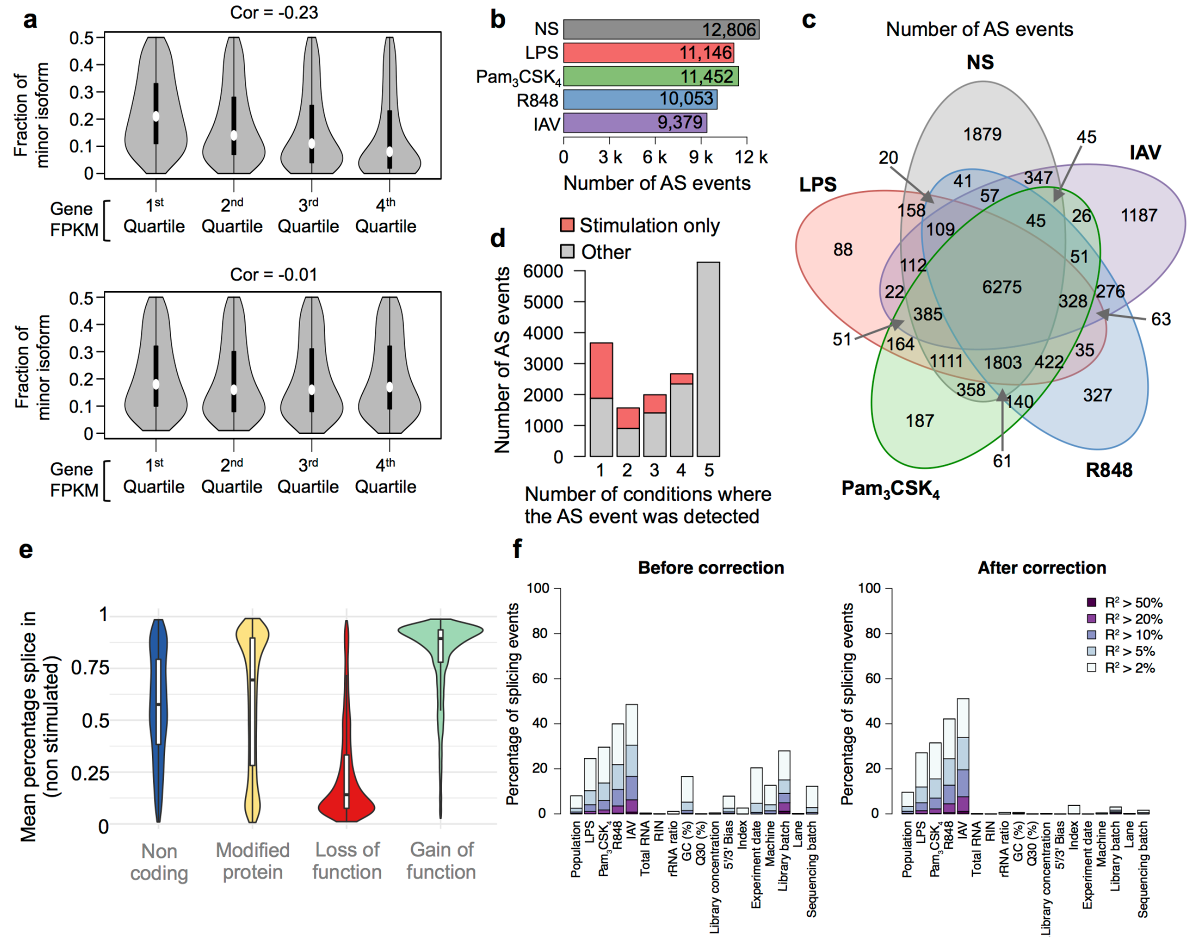

Supplementary Figure 2 | Quantification and normalisation of alternative splicing (AS) variability upon stimulation.

a Distribution of the fraction of minor isoforms as a function of gene FPKM, when considering all AS events (upper panel) or the subset of AS events for which the global gene expression is higher than 10 FPKM and the minor isoforms account for more than 5% of total gene transcripts (lower panel). **b** Number of AS events detected in the non-stimulated state and within each stimulation condition. Colour code reflects the experimental condition **c** Number of AS events detected for each combination of conditions. **d** Sharing of AS events across conditions. AS events that are detected specifically after stimulation are highlighted in red. **e** Distribution of PSI values in the non-stimulated state according to their putative consequence at protein level (centre line, median; box limits, upper and lower quartiles; whiskers, 1.5x interquartile range). Colours reflect consequence of AS at the protein level. **f** Effect of technical batches and confounding factors on inferred splicing levels. The proportion of AS events that are associated with each factor is presented, for different levels of explained variance (R^2), before and after correction of the data (left and right panels, respectively). For every AS event, the proportion of the variance in PSI values explained by each cofactor was assessed through a linear mixed model adjusting for all other cofactors under consideration.

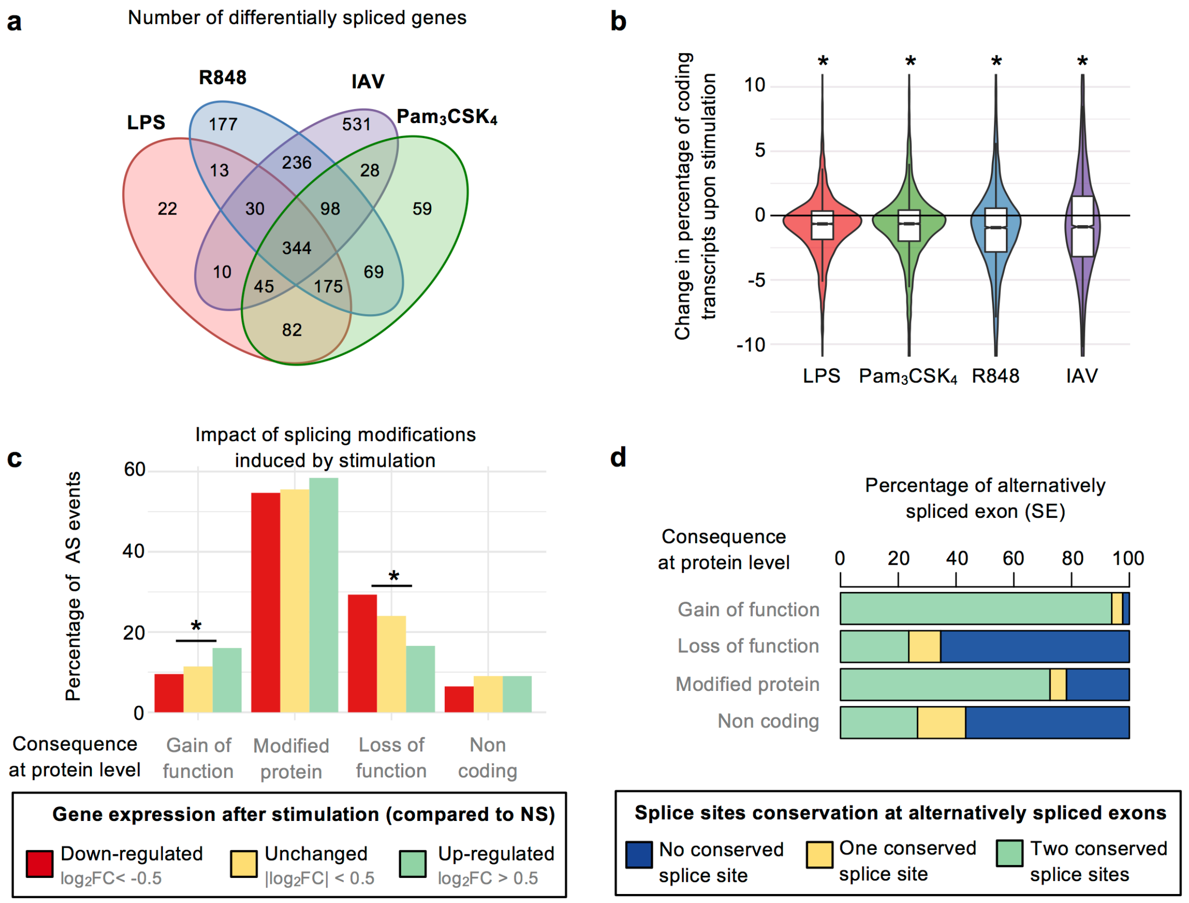

Supplementary Figure 3 | Putative coding consequences of changes in alternative splicing upon stimulation.
a Context-specificity of changes in AS upon stimulation. For each set of stimulation conditions, we report the number of genes that are differentially spliced in these conditions compared to the non-stimulated state. **b** Change in percentage of coding transcripts upon stimulation. For each stimulus, violin plot show the distribution, across all loss/gain of function AS events, of the difference in PSI values between the stimulated and non stimulated state, multiplied by 1 for gain of function events and -1 for loss of function events (* Wilcoxon $p < 1.1 \times 10^{-16}$; centre line, median; box limits, upper and lower quartiles; whiskers, 1.5x interquartile range). Colours reflect the experimental condition. **c** Predicted consequences of changes in AS upon stimulation, according to the maximal fold change in expression. (Fischer's exact test for equal proportions, *$p < 0.001$). **d** Conservation at splice sites of alternatively spliced exons, according to the putative consequences of exon inclusion at the protein level.

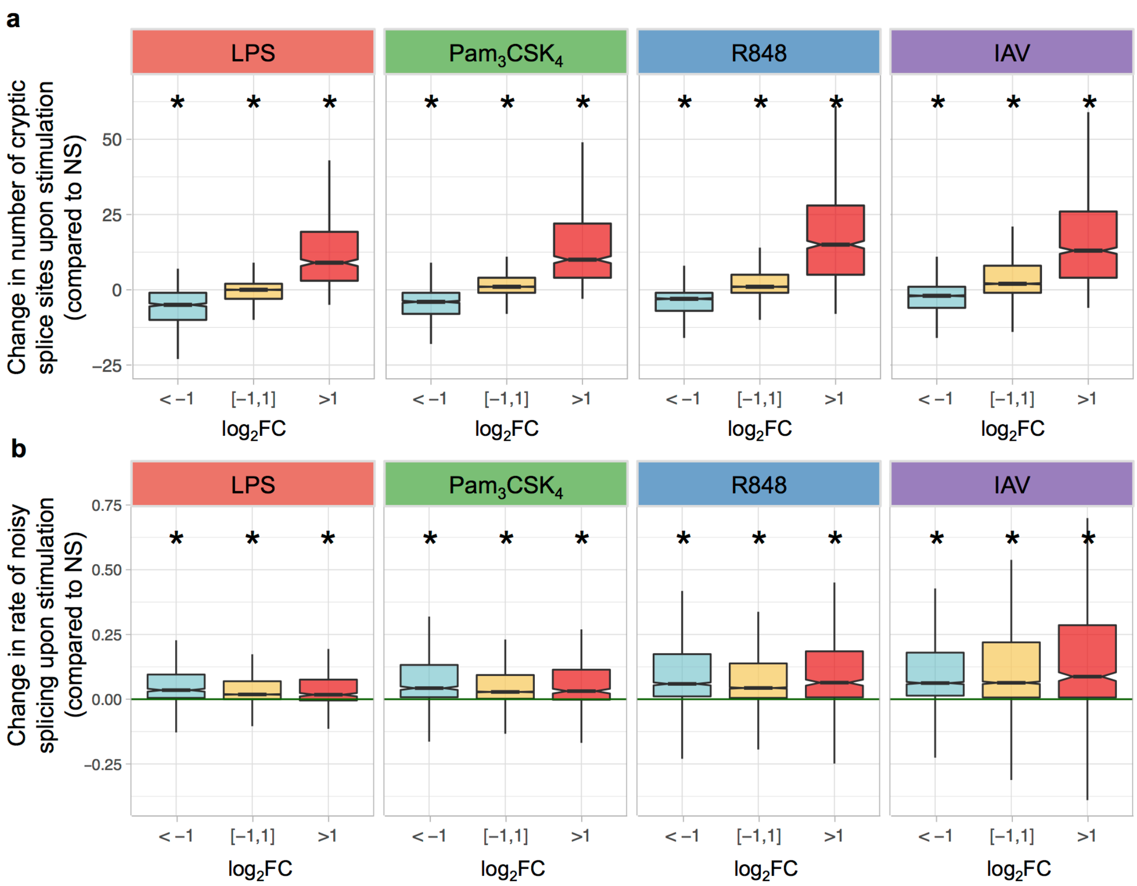

Supplementary Figure 4 | Detection of cryptic splice sites and change in rate of noisy splicing as a function of fold changes in gene expression.
For each stimulus, genes are grouped according to their fold change in expression upon stimulation ($\log_2$FC). The distribution of (**a**) the change in number of cryptic splice sites detected per gene between the stimulated and non-stimulated state, and (**b**) the change in mean rate of noisy splicing per gene between the stimulated and non-stimulated state are reported (* Wilcoxon $p < 10^{-10}$). Boxes are coloured according to their $\log_2$FC. (blue: $\log_2$FC< -1 ; yellow -1<$\log_2$FC<1; red $\log_2$FC>1; centre line, median; box limits, upper and lower quartiles; whiskers, 1.5x interquartile range)

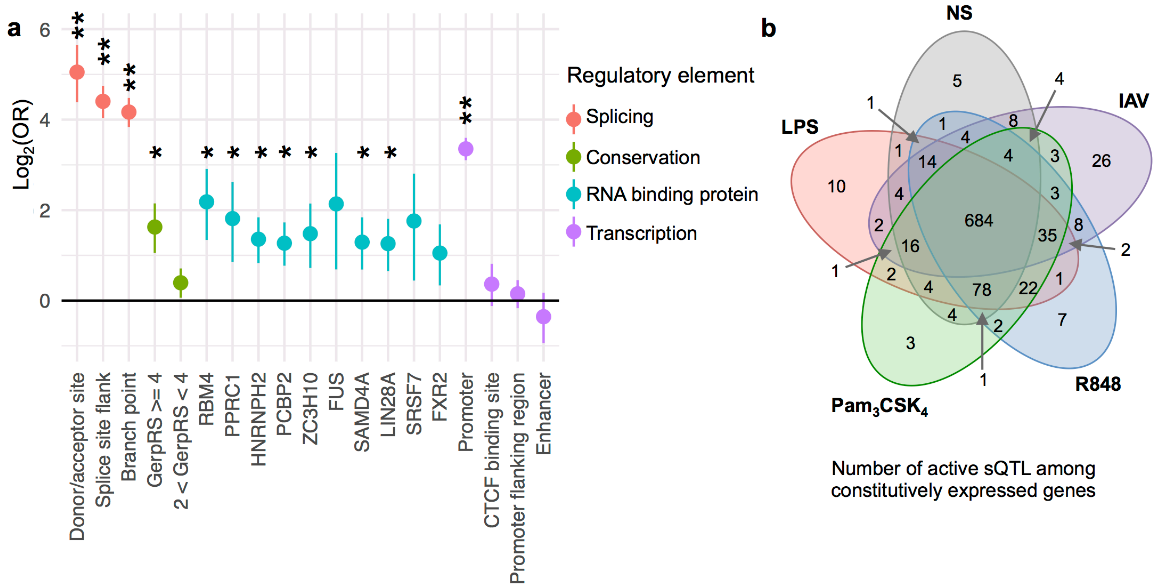

Supplementary Figure 5 | Genetic bases of alternative splicing.
a. Enrichment of regulatory features at sQTLs compared to genome-wide expectations (Fisher exact test; *:$p_{adj} < 0.05$, **: $p_{adj} < 1.1\times10^{-16}$). For sites bound by RNA binding proteins (RBPs), only the 10 most significant RBPs are shown. Error bars indicate 95% confidence intervals. **b**. Sharing of sQTLs among constitutively expressed genes (FPKM > 10 in all conditions). For each sQTL, sharing is assessed by comparing all possible models of sharing and retaining the model with the highest likelihood (**Methods; Supplementary Note 4**).

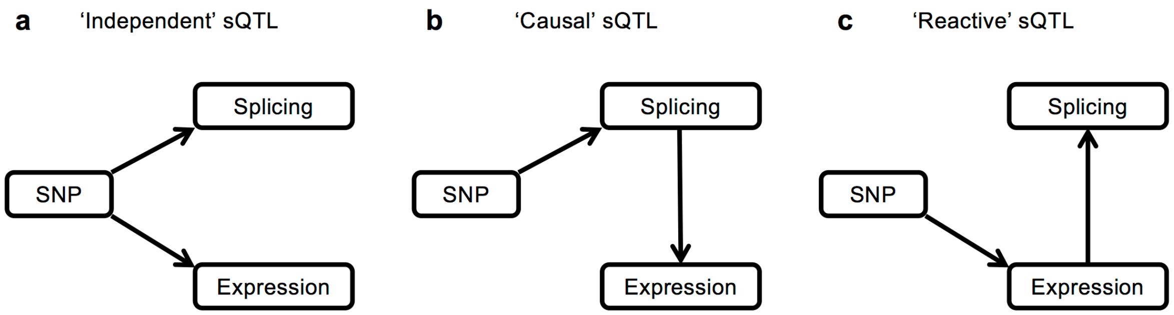

Supplementary Figure 6 | Possible models tested in our Likelihood-based Causal Model Selection framework.
For each model, black arrows indicate possible causal relationships between genetics (SNPs), splicing and gene expression. To assign causality, we first excluded independent sQTLs (**a**) by testing whether splicing is independent from expression after adjusting for genotype, and then compared the likelihood of the remaining models (**b**) and (**c**).

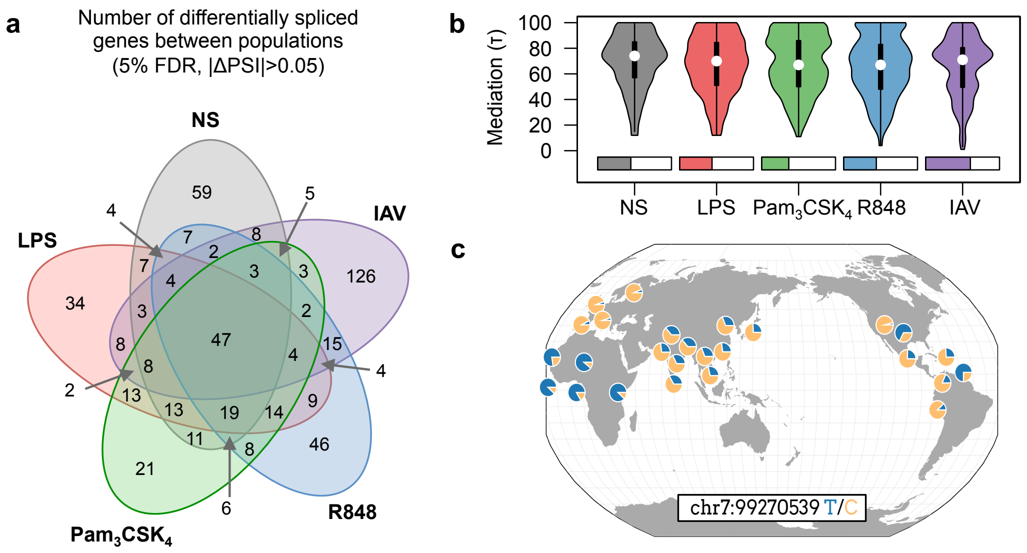

Supplementary Figure 7 | Population differences in alternative splicing.
a Sharing of differentially spliced genes between populations (pop-DSG) across conditions. **b** Percentage of population differences in AS that is attributable to genetic variants. For each condition, the violin plot shows the distribution, across AS events with a sQTL, of the percentage of population differences mediated by genetics (white dot, median; box limits, upper and lower quartiles; whiskers, 1.5x interquartile range). Horizontal bars indicate the percentage of AS events that do not have a sQTL (corresponding to a mediation effect of 0), among AS events that are different between populations. **c** Geographic distribution of allelic frequencies of the *CYP3A5* sQTL rs776746.

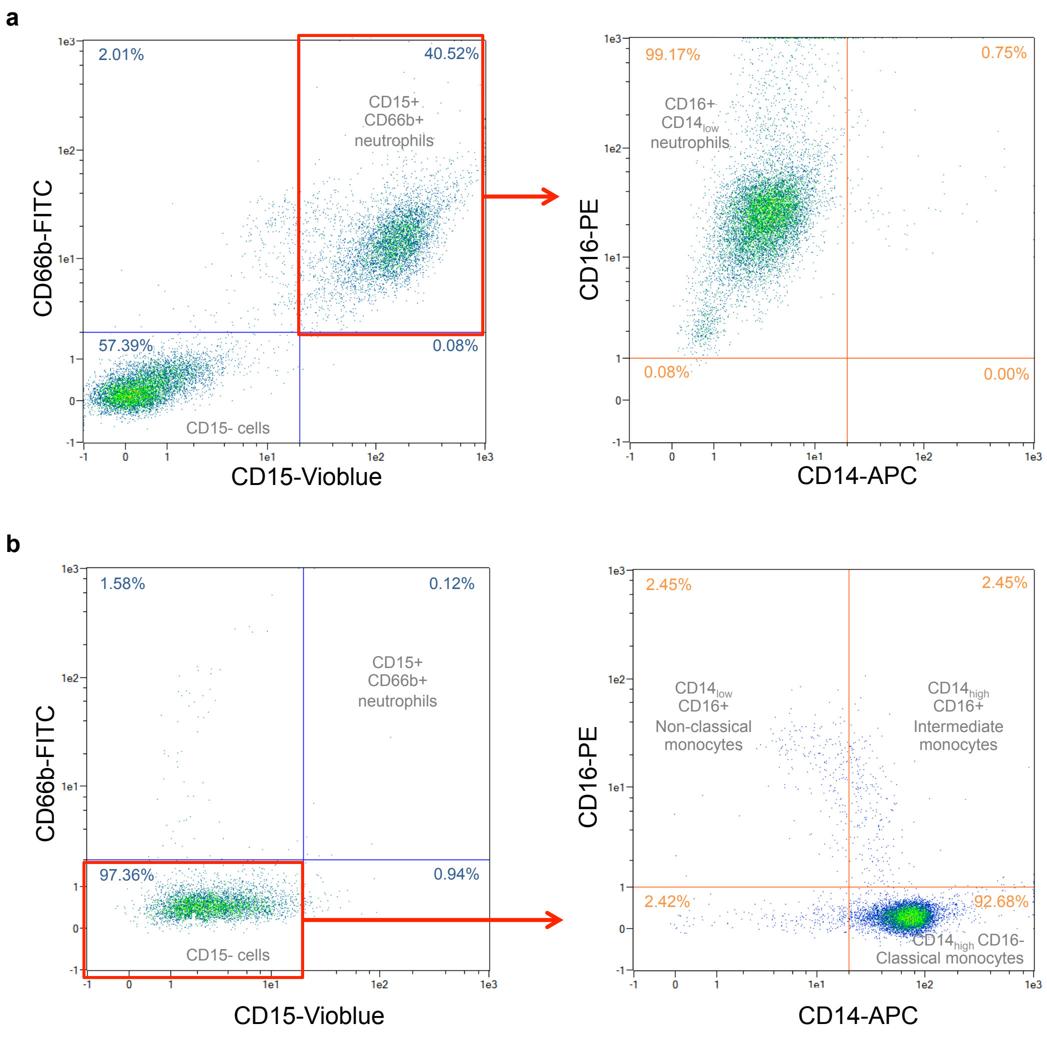

Supplementary Figure 8 | Characterisation of neutrophils and monocytes by flow cytometry.

a Flow cytometry analysis on the granulocyte/erythrocyte layer obtained from a whole blood density gradient centrifugation. The labeling of the sample by four antibodies showed that neutrophils are $CD15^+$, $CD66b^+$ (left panel), $CD16^+$ and $CD14^{low}$ (right panel). **b** Flow cytometry analysis on primary monocytes obtained using the same experimental settings as in Quach et *al*. 2016. The labeling of the sample revealed that neutrophils ($CD15^+$/$CD66b^+$) were virtually absent from the monocyte cell fraction (0.12%, left panel). The $CD15^-$/$CD66b^-$ cells corresponded to the three monocytes subtype: $CD14^{high}$/$CD16^-$ classical monocytes (92.7%), $CD14^{high}$/$CD16^+$ intermediate monocytes (2.5%), and $CD14^{low}$/$CD16^+$ non-classical monocytes (2.5%, right panel).

Supplementary References

1. Blanchette, M. *et al.* Aligning multiple genomic sequences with the threaded blockset aligner. *Genome Res* **14**, 708-715 (2004).
2. Sagulenko, P., Puller, V. & Neher, R.A. TreeTime: Maximum-likelihood phylodynamic analysis. *Virus Evol* **4**, vex042 (2018).
3. Kumar, S. & Hedges, S.B. A molecular timescale for vertebrate evolution. *Nature* **392**, 917-920 (1998).
4. Leek, J.T., Johnson, W.E., Parker, H.S., Jaffe, A.E. & Storey, J.D. The sva package for removing batch effects and other unwanted variation in high-throughput experiments. *Bioinformatics* **28**, 882-883 (2012).
5. Schadt, E.E. *et al.* An integrative genomics approach to infer causal associations between gene expression and disease. *Nat Genet* **37**, 710-717 (2005).
6. Prufer, K. *et al.* The complete genome sequence of a Neanderthal from the Altai Mountains. *Nature* **505**, 43-49 (2014).
7. Quach, H. *et al.* Genetic Adaptation and Neandertal Admixture Shaped the Immune System of Human Populations. *Cell* **167**, 643-656 e617 (2016).
8. 1,000 Genomes Project Consortium. A global reference for human genetic variation. *Nature* **526**, 68-74 (2015).
9. Sankararaman, S. *et al.* The genomic landscape of Neanderthal ancestry in present-day humans. *Nature* **507**, 354-357 (2014).
