# Supplementary material for: Defining the genetic and evolutionary architecture of alternative splicing in response to infection
Source: Nat Commun. 2019 Apr 11;10:1671. doi: 10.1038/s41467-019-09689-7 (PMC6459842; doi:10.1038/s41467-019-09689-7)
Supplement: Supplementary file 3 — Reporting Summary [file 41467_2019_9689_MOESM3_ESM.pdf]

nature research

Corresponding author(s): Lluis Quintana-Murci

Last updated by author(s): Mar 20, 2019

Reporting Summary

Nature Research wishes to improve the reproducibility of the work that we publish. This form provides structure for consistency and transparency in reporting. For further information on Nature Research policies, see Authors & Referees and the Editorial Policy Checklist.

Statistics

For all statistical analyses, confirm that the following items are present in the figure legend, table legend, main text, or Methods section.

n/a	Confirmed	
☐	☒	The exact sample size (n) for each experimental group/condition, given as a discrete number and unit of measurement
☐	☒	A statement on whether measurements were taken from distinct samples or whether the same sample was measured repeatedly
☐	☒	The statistical test(s) used AND whether they are one- or two-sided<br>*Only common tests should be described solely by name; describe more complex techniques in the Methods section.*
☐	☒	A description of all covariates tested
☐	☒	A description of any assumptions or corrections, such as tests of normality and adjustment for multiple comparisons
☐	☒	A full description of the statistical parameters including central tendency (e.g. means) or other basic estimates (e.g. regression coefficient) AND variation (e.g. standard deviation) or associated estimates of uncertainty (e.g. confidence intervals)
☐	☒	For null hypothesis testing, the test statistic (e.g. F, t, r) with confidence intervals, effect sizes, degrees of freedom and P value noted<br>*Give P values as exact values whenever suitable.*
☒	☐	For Bayesian analysis, information on the choice of priors and Markov chain Monte Carlo settings
☒	☐	For hierarchical and complex designs, identification of the appropriate level for tests and full reporting of outcomes
☐	☒	Estimates of effect sizes (e.g. Cohen's d, Pearson's r), indicating how they were calculated

Our web collection on statistics for biologists contains articles on many of the points above.

Software and code

Policy information about availability of computer code

Data collection	For gene expression levels, RNA reads were aligned to the human genome with TopHat (Kim et al., Genome Biol., 2013), QC metrics were computed using RseqQC package (Wang et al., Bioinformatics, 2012), and quantification of FPKM was performed using Cufflinks/CuffDiff (v2.0.2) (Trapnell et al., Nat. Protoc., 2012).<br><br>For splicing events, RNA reads were re-aligned to the human genome with HISAT2 and junction reads were quantified using leafcutter (Li et al., Nat. Genet., 2018). Known AS events were annotated using eventGenerator script of SUPPA (Alamancos et al., RNA, 2015) and PSI values were quantified using MISO (Katz et al., Nat methods, 2010). Missing values were imputed using impute.knn function from the impute package, and adjusted for batch effects using ComBat (Johnson et al., Biostatistics, 2007)<br><br>For genotypes, PLINK v1.9 (Chang et al., Gigascience, 2015) was used for QC and SNP filtering. For whole-exome sequencing, reads pairs were mapped with BWA v.0.7.7 (Li and Durbin, Bioinformatics, 2009), and duplicate reads were marked with Picard Tools v.1.94. Genotype calls were then made with GATK v.3.2.2 (DePristo et al., Nat. Genet., 2011), according to the GATK Best Practice recommendations. Before imputation, we phased the data with SHAPEIT2 (Delaneau et al., Nat. Methods, 2013), using 500 conditioning haplotypes, 50 MCMC iterations, 10 burnin and 10 pruning iterations. Genotype imputation was performed with IMPUTE v.2 (Howie et al., PloS Genet., 2009), considering 1-Mb windows and a buffer region of 1 Mb.
Data analysis	Binding sites of RNA binding proteins were prediceted using HOMER (Heinz, Mol Cell ,2010)<br><br>All other analyses were performed in R/3.5.0 or Python/2.7.8.<br>Scripts are available on github under MIT License (https://github.com/mrotival/EvoImmunoPop_Splicing)

For manuscripts utilizing custom algorithms or software that are central to the research but not yet described in published literature, software must be made available to editors/reviewers. We strongly encourage code deposition in a community repository (e.g. GitHub). See the Nature Research guidelines for submitting code & software for further information.

Data

Policy information about availability of data

All manuscripts must include a data availability statement. This statement should provide the following information, where applicable:

- Accession codes, unique identifiers, or web links for publicly available datasets
- A list of figures that have associated raw data
- A description of any restrictions on data availability

Genome-wide SNP genotyping, whole exome sequencing and RNA-sequencing data used in this study have been deposited in the European Genome-phenome Archive (EGA) under accession code EGA: EGAS00001001895. Source data for figure(s) 1-6 are provided with the paper. All other relevant data is available upon request.

Field-specific reporting

Please select the one below that is the best fit for your research. If you are not sure, read the appropriate sections before making your selection.

☒ Life sciences ☐ Behavioural & social sciences ☐ Ecological, evolutionary & environmental sciences

For a reference copy of the document with all sections, see nature.com/documents/nr-reporting-summary-flat.pdf

Life sciences study design

All studies must disclose on these points even when the disclosure is negative.

Sample size	Sample size of 100 individuals per population and condition was determined based on sample size of previous eQTL studies (Pickrell , Nature, 2010) and to ensure ~90% power for the detection of eQTLs that account for 20% or more of the variance in gene expression, when using a genome-wide threshold of 5×10-8.
Data exclusions	Stimulation experiments were performed on 200 individuals, for a target number of 1000 samples (200×5 conditions). Out of these samples, 22 were excluded prior to library preparation due to low concentration of RNA (total mass < 2.5 mg) or bad RNA quality (RIN < 7). Furthermore, 8 samples were excluded after sequencing due to irregular gene body coverage (total area under GeneBodyCoverage curve < 0.8).
Replication	The reproducibility of RNA-Seq profiles was evaluated by technical and biological replicates on seven independent individuals (4 Africans and 3 Europeans) across the five experimental conditions.
Randomization	We used a balanced designed where each experimental batch was composed of three randomly selected individuals of European-descent and three randomly selected individuals of African-descent. Samples of stimulated and resting monocytes were then pooled together across all batches and randomized prior to library preparation. Sequencing libraries where prepared by group of ~12 randomly selected samples, and samples were pooled by group of 6 within each lane (3 randomly selected Europeans and Africans).
Blinding	When performing stimulations, experimentators were blinded to the population of origin of the individual, within each experimental batch. Genotypes were unknown a priori during data collection and randomized by meiotic recombination. Sequencing and quantification of AS events were done through automated pipelines and did not take into account the identifty of the sample or population of origin.

Reporting for specific materials, systems and methods

We require information from authors about some types of materials, experimental systems and methods used in many studies. Here, indicate whether each material, system or method listed is relevant to your study. If you are not sure if a list item applies to your research, read the appropriate section before selecting a response.

Materials & experimental systems

n/a	Involved in the study
☒	☐ Antibodies
☒	☐ Eukaryotic cell lines
☒	☐ Palaeontology
☒	☐ Animals and other organisms
☐	☒ Human research participants
☒	☐ Clinical data

Methods

n/a	Involved in the study
☒	☐ ChIP-seq
☐	☒ Flow cytometry
☒	☐ MRI-based neuroimaging

Human research participants

Policy information about studies involving human research participants

Population characteristics	The studied population was composed of 100 male donors of self-reported European descent (EUB) and 100 of self-reported African descent (AFB), all living in Belgium. Inclusion was restricted to donors between 19 and 50 years of age, nominally healthy at the time of sample collection. Serological testing was performed for all donors, and those with serological signs of past or ongoing infection with human immunodeficiency virus (HIV), hepatitis B virus (HBV) or hepatitis C virus (HCV) were excluded. No overrepresentation of any particular disease was observed relative to official report statistics published by the World Health Organization or in epidemiological studies.
Recruitment	Recruitment was done at the Center for Vaccinology (CEVAC) of Ghent University Hospital (Ghent, Belgium), based on self reported ancestry.
Ethics oversight	Samples were collected after written informed consent had been obtained, and the study was approved by the local ethics committee (Ethics Committee of the Ghent University), the Ethics Board of Institut Pasteur (EVOIMMUNOPOP-281297) and the relevant French authorities (CPP, CCITRS and CNIL).

Note that full information on the approval of the study protocol must also be provided in the manuscript.

Flow Cytometry

Plots

Confirm that:

☒ The axis labels state the marker and fluorochrome used (e.g. CD4-FITC).

☒ The axis scales are clearly visible. Include numbers along axes only for bottom left plot of group (a 'group' is an analysis of identical markers).

☒ All plots are contour plots with outliers or pseudocolor plots.

☒ A numerical value for number of cells or percentage (with statistics) is provided.

Methodology

Sample preparation	Monocytes were positively selected with magnetic CD14 microbeads, according to the manufacturer's instructions. Neutrophils were collected from the granulocyte/erythrocyte layer obtained by performing a Ficoll-Paque density gradient.
Instrument	MACSQuant cytometer from Miltenyi
Software	MACSQuantify version 2.11
Cell population abundance	In the granulocyte/erythrocyte layer, 40.5% of cells were defined as CD15+ CD66b+ CD16+ and CD14low neutrophils. In the monocyte fraction, we identified three subtypes: the CD14high CD16- classical monocytes (92.7%), the CD14high CD16+ intermediate monocytes (2.5%) and the CD14low CD16+ non-classical monocytes.
Gating strategy	Neutrophils were defined on the basis of CD15 and CD66b markers, whereas CD14 and CD16 markers were used to characterize monocyte subtypes.

☒ Tick this box to confirm that a figure exemplifying the gating strategy is provided in the Supplementary Information.
