# Supplementary material for: Defining the genetic and evolutionary architecture of alternative splicing in response to infection
Source: Nat Commun. 2019 Apr 11;10:1671. doi: 10.1038/s41467-019-09689-7 (PMC6459842; doi:10.1038/s41467-019-09689-7)
Supplement: Supplementary file 4 — Description of Additional Supplementary Files [file 41467_2019_9689_MOESM4_ESM.pdf]

Description of Additional Supplementary Files

Supplementary Data 1: Characterisation of non-conserved and recent splice sites
a GO enrichment analysis of non-conserved splice sites. **b** Enrichment of non-conserved splice sites in stimulation-induced genes. **c** List of human-specific splice sites.

Supplementary Data 2: Changes in alternative splicing events upon stimulation
a Description of alternative splicing (AS) events used in this study. **b** Changes in AS upon stimulation. **c** Comparison of differentially spliced and differentially expressed genes in response to stimulation. **d** Levels of noisy splicing per gene.

Supplementary Data 3: Genetic bases of alternative splicing variability in resting and stimulated monocytes
a List of detected sQTLs. **b** GO enrichments of genes with a sQTL. **c** Overlap of sQTLs with eQTLs. **d** Stimulation-specific sQTLs. **e** Overlap of sQTLs with GWAS loci.

Supplementary Data 4: Analysis and sources of population differences in alternative splicing **a** Population differences in alternative splicing. **b** Signals of positive selection based on empirical F_{ST} outliers. **c** Signals of positive selection based on empirical |iHS| outliers (Africans). **d** Signals of positive selection based on empirical |iHS| outliers (Europeans). **e** List of archaic sQTLs. **f** Enrichment of archaic sQTLs according to the threshold used for the calling of Neanderthal haplotypes.
